# The relationship between harsh parenting, shyness and cyber victimization: based on the cross-lagged panel model and the random intercept cross-lagged panel model

**DOI:** 10.3389/fpsyt.2025.1607367

**Published:** 2025-06-27

**Authors:** Xintong Zhao, Tao Xin, Qingsong Sang

**Affiliations:** ^1^ School of Educational Science, Anhui Normal University, Wuhu, Anhui, China; ^2^ Collaborative Innovation Center of Assessment toward Basic Education Quality, Beijing Normal University, Beijing, China

**Keywords:** harsh parenting, shyness, cyber victimization, CLPM, RI-CLPM

## Abstract

**Objective:**

To examine the longitudinal associations between harsh parenting, shyness, and cyber victimization. The present study hypothesized that shyness mediated harsh parenting and cyber victimization.

**Methods:**

Eight hundred and twenty-eight first-year middle school students voluntarily completed three rounds of questionnaires investigating harsh parenting, shyness, and cyber victimization at eight-month intervals. CLPM and RI-CLPM were conducted using SPSS 26.0 and Mplus 8.3.

**Results:**

Shyness was a significant predictor of cyber victimization and harsh parenting had a significant prospective effect on cyber victimization. Shyness mediated longitudinally between harsh parenting and cyber victimization.

**Conclusions:**

In CLPM, T1 and T2 harsh parenting significantly and positively predicted T3 cyber victimization, with shyness serving as a mediator in the longitudinal relationship between harsh parenting and cyber victimization. In RI-CLPM, at the within-individual level, T1 harsh parenting significantly and positively predicted T2 cyber victimization, T2 shyness mediated the relationship between T1 harsh parenting and T3 cyber victimization. This study deepens our understanding of the dynamic relationship among harsh parenting, shyness, and cyber victimization, providing robust empirical evidence and meaningful insights for interventions in adolescent cyber victimization.

## 1 Introduction

In the digital era, the Internet has become the “second battlefield” for psychological development. Along with the rapid growth in the number of Internet users, the combination of traditional victimization and the Internet has generated cyber victimization (1). Unlike traditional victimization, which relies on physical contact, cyber victimization has increasingly become the focus of researchers' attention due to its anonymity and lack of temporal and spatial constraints (2). Specifically, cyber victimization refers to individuals who are repeatedly subjected to malicious and aggressive behaviors from other individuals or groups during electronic information exchange (3), including forms of verbal intimidation, insults, verbal abuse, and malicious harassment (4). Individuals who are chronically victimized online often suffer from a range of internalizing and externalizing problems, such as anxiety and depression (5), which may even be severe enough to led to suicidal behavior (6). These problems can have a significant negative impact on the mental health of the victim. Some studies have pointed out that adolescents are being subconsciously shaped by the online environment, and the Internet is gradually becoming an important way for adolescents to address social developmental issues (7). Life course theory posits that if adolescents' developmental problems in one area (e.g., cyber victimization) are not effectively addressed during a critical transition period in their development (e.g., when they first enter middle school), it may trigger a chain reaction of events that could have serious consequences in many areas in the future (8). Middle school, as a critical developmental transition for adolescents, coincides with heightened susceptibility to the overwhelming volume of online information. According to Tokunaga (9), adolescence is a period of high prevalence of cyber victimization, and the trend of cyber victimization follows a curvilinear pattern as the age of the adolescent increases, with the highest prevalence rates occurring among middle school students in the seventh and eighth grades (approximately 13–15 years of age). In a study of middle school students in the United States, it was found that about 11 percent of students reported experiencing cyber victimization at least once in the past few months (10). In addition, a keyword clustering study found a significant association between “cyber victimization” and “adolescents” (11). Furthermore, Zhang et al. (12) found that 53.5% of middle school students in China reported having experienced cyber victimization. This data reveals the severity of cyber victimization among adolescents in China. Therefore, it is of great theoretical and practical significance to study in depth the role mechanisms of cyber victimization among middle school students.

From the integrated child development model, it can be seen that both external factors (e.g., parental phubbing) and internal factors (e.g., anxiety) can have an impact on adolescents' cyber victimization (13–15). Current research on factors influencing adolescent cyber victimization focuses on adolescents' own mobile phone use behaviors and lacks attention to parenting styles (16). Among many types of parenting styles, harsh parenting covers a wide range of manifestations and is not identical to other parenting behaviors, although there is some overlap. Researchers have argued that there is a need to examine harsh parenting as a distinct style of parenting, which would not only reveal its negative impact on child development, but also allow for a broader examination of the consequences of harsh parenting (17). Harsh parenting is a negative and neglectful parenting style that includes three aspects of harsh behaviors, harsh emotions, and harsh attitudes, mainly including physical aggression, verbal aggression, psychological aggression, and controlling behaviors (18). It is not only manifested in outwardly violent behaviors such as yelling and beating at children (19), but also in internal emotional attitudes such as neglecting, opposing, rejecting, and threatening children (20). On the one hand, according to social learning theory, adolescents who receive violent treatment from their parents are more tolerant of violent behavior (21). Research has found that harsh parenting can result in adolescents feeling neglect and rejection from their parents when they are cyber victimized (22), this may make adolescents reluctant to turn to their parents even when they are cyber victimized, leading to more cyber victimization among adolescents. Additionally, Lereya et al. (23) implemented a meta-analysis of 70 studies, demonstrating that negative parenting styles positively correlate with cyber victimization. Drawing on these studies, it can be inferred that harsh parenting may serve as a significant positive predictor of adolescents' cyber victimization.

However, this relationship is bidirectional, cyber victimization may also be reactive to harsh parenting. Parental acceptance and rejection theory emphasizes that the family is a dynamic and interactive system in which parents and children's behaviors interact and influence each other's (18). This relationship is reflected not only in the interactions between parenting behaviors and the child's growth, but also in the fact that the child's developmental characteristics trigger different parental responses over time, which may in turn be counterproductive to the child's subsequent development (24). In addition, developmental contextualism posits that there is a dynamic interplay between the individual and the multiple environments in which they exist (25). Empirical studies have found that parenting style is a significant predictor of cyber victimization (26). Harsh parenting is strongly associated with adolescent victimization (27). Adolescents who suffer from cyber victimization can suffer from a range of problematic behaviors such as depression and anxiety (5). When parents recognize these adverse effects, they may exert psychological control over their children and limit their behaviors (28). Furthermore, given that Chinese parents tend to have high academic expectations for their children (29). When they perceive the negative impacts of adolescents experiencing cyber victimization, they may engage in some controlling behaviors towards their adolescents for the purpose of promoting their academic development (30), which may exacerbate the likelihood of harsh parenting occurring. Consequently, there may be an interaction mechanism between harsh parenting and cyber victimization among middle school students.

Shyness refers to an individual's discomfort or behavioral inhibition in social situations, usually stemming from a fear of negative evaluations, which is often accompanied by emotional frustration or inhibition and can significantly affect individuals' willingness to engage in activities as well as their personal pursuits (31). Attachment theory posits that individuals experiencing harsh parenting tend to develop maladaptive affective-cognitive associations and negative self-schemas, leading to withdrawal behaviors that foster shyness development (32). Empirical research has demonstrated that children who are frequently reprimanded and physically punished tend to exhibit increased negative evaluations of their emotions and higher levels of shyness (33). Harsh parenting can intensify children's shyness, resulting in more frequent negative emotional experiences and a tendency toward negative interpretations of social situations (34). Shy individuals can exhibit introverted and neurotic traits that are highly similar to the victim's personality traits (35, 36). In addition, according to the social adaptation model, shy individuals have negative perceptions of the environment and others, and will negatively interpret behaviors from others (37). Therefore, in online environments, shy individuals may view the behavior of others as rejection or harm to themselves and consider themselves as experiencing cyber victimization. Secondly, shy individuals may choose to avoid and allocate fewer attentional resources after they have noticed threats (38), which may lead them to choose avoidance when they face threats in the network, exacerbating the threats posed to them by others. And finally, shy individuals lack sufficient social support, and they tend to seek less help from others when experiencing cyber victimization (39), which also increases their risk of experiencing cyber victimization to a certain extent.

However, upon a comprehensive review of the existing literature, we have identified certain deficiencies. Firstly, the majority of studies have utilized a cross-sectional design (40–43). This design approach poses challenges in effectively capturing the dynamic process of change among variables and often lacks a thorough exploration of the longitudinal relationships among these variables. Secondly, previous research has insufficiently differentiated between “between-individual” and “within-individual” effects, potentially resulting in biased estimations of between-variable relationships (44). Furthermore, while prior studies have explored the relationship between harsh parenting and adolescent psychological issues, few have specifically investigated its direct relationship with cyber victimization using longitudinal approaches. In light of the above, the current study adopted a longitudinal tracking design. Through the integration of the cross-lagged panel model (CLPM) and the random intercept cross-lagged panel model (RI-CLPM), we seeks to uncover the underlying mechanisms between harsh parenting and adolescent cyber victimization, offering a stronger basis for understanding its pathways.

In previous studies, interactions between variables have usually been analyzed through the CLPM model, a method that mainly reveals the relationships between the trait-based components of variables. However, the crux of developmental theory is to explore the relationships between the stateful components of variables at the within-individual level (45). Therefore, the use of traditional CLPM models may result in a degree of mismatch between developmental theory and the statistical analysis models used to test the theoretical hypotheses (46). The traditional CLPM model has limitations, which are mainly reflected in the inability to effectively separate between-individual differences from within-individual changes, and its analytical results mainly focus on the decomposed between-person effects (47). As time passes, individual characteristics fluctuate compared to their initial levels, and this within-individual variation is more conducive to inferring causality of variables over time. In light of this, the researchers proposed the RI-CLPM model. RI-CLPM distinguishes between-individual differences through random intercepts and captures within-individual dynamics via time-varying residuals, thereby more accurately revealing the temporal associations between variables (44). By separating the between-individual variance from the within-individual variance, RI-CLPM can present a clearer picture of the dynamic process of individual change in a time series (44), this improvement significantly enhances the explanatory power of the model for the within-individual effects and provides a more robust framework for longitudinal data analysis (47).

Despite the fact that CLPM and RI-CLPM possess distinct analytical strengths, they are complementary rather than mutually exclusive in examining temporal dynamics between variables. Methodological research suggests that integrating both models offers a comprehensive analytical framework, enabling simultaneous examination of group-level associations and within-individual change mechanisms, thereby providing a more complete understanding of variable interactions (47, 48). Based on this, the present study used both CLPM and RI-CLPM methods to comprehensively examine the interaction between harsh parenting and cyber victimization so as to gain a deeper understanding of the longitudinal relationship.

## 2 Materials and methods

### 2.1 Participants

A cluster sampling method was utilized to select first-year middle school students from four middle schools in Anhui Province. The study conducted three survey waves, with questionnaires administered in classroom settings under teacher supervision. All participants provided written informed consent and voluntarily participated in this anonymous study.

Three measurements were taken in January 2023 (T1), September 2023 (T2), and May 2024 (T3), with an interval of eight months each time. The criteria for valid questionnaires were no regularity of responses and repetitions, no missing information, reasonable response time, and no logical conflict before or after the questionnaire was done. A total of 903 valid questionnaires were collected in the first time, 853 valid questionnaires were obtained in the second time, and by the end of the third time, a total of 75 participants had been lost due to class transfer or other reasons. A total of 828 valid questionnaires were obtained and the loss rate of questionnaires was 8.3%. Among them, 403 (48.7%) were boys and 425 (51.3%) were girls, 518 (62.6%) were urban and 310 (37.4%) were rural. The mean age of the subjects at the first time was 12.13 ± 1.21.

The results of the independent samples *t*-test showed that there were no significant differences between the 75 participants and the 828 valid participants on harsh parenting [*t* (828) = 0.65, *p*>0.05], shyness [*t*(828) = -0.47, *p*>0.05], and cyber victimization [*t*(828) = 0.82, *p*>0.05 ], suggesting that there was no structured attrition of the participants.

### 2.2 Methods

#### 2.2.1 Harsh parenting scale

The Harsh Parenting Scale originally developed by Simon et al. (49) and subsequently revised by Wang (50) was employed in this study. This scale comprises four items and is scored using a 5-point Likert scale ranging from 1 (not at all compliant) to 5 (fully compliant). Higher scores on this scale indicate greater exposure to harsh parenting behaviors. The Cronbach's alpha coefficients for the three measurements of the scale were 0.918, 0.925, and 0.923, respectively.

#### 2.2.2 Shyness scale

The Shyness Scale, developed by Cheek and Buss (51), was utilized in this study to evaluate middle school students' behavioral inhibitions in social situations and their self-assessment of social behavior. The questionnaire is one-dimensional, with 13 items, and is scored on a 5-point scale from 1 (not at all compliant) to 5 (fully compliant), with higher scores indicating higher levels of shyness. The scale has been tested by Chinese scholars with good reliability and validity (52), and has been widely used in the measurement of Chinese student groups (53, 54). Across the three measurement waves, the scale exhibited high internal consistency, with Cronbach's alpha coefficients of 0.977, 0.978, and 0.974, respectively.

#### 2.2.3 Cyber victimization scale

It was a subscale of the Cyberbullying Scale, which was originally developed by Topcu and Erdur-Baker (55) and then subsequently translated by Chinese scholars Chu and Fan (56). This subscale consists of 14 items, each rated on a 4-point Likert scale ranging from 1 (never perpetrated) to 4 (more than 3 times). The scale demonstrated high internal consistency across the three measurement waves, with Cronbach's alpha coefficients of 0.976, 0.978, and 0.980, respectively.

### 2.3 Data analysis

This study used SPSS 26.0 and Mplus 8.3 for data analysis. SPSS 26.0 was utilized to conduct descriptive statistics and correlation analysis. Longitudinal measurement invariance was tested using Mplus 8.3, followed by analyses of CLPM and RI-CLPM. Additionally, the mediating role of shyness in the relationship between harsh parenting and cyber victimization was examined, the mediating effect was tested using the Bootstrap method.

## 3 Results

### 3.1 Common method bias test

Given that the data were derived from self-reports, Harman single-factor test was conducted for all variables. All items related to harsh parenting (T1, T2, T3), shyness (T1, T2, T3), and cyber victimization (T1, T2, T3) were included in Harman single-factor test (53). The results indicated that nine factors with eigenvalues greater than 1 were extracted, with the first factor accounting for 24.221% of the variance, which is below the critical threshold of 40%, this suggests that common method bias is not a significant concern in this study (54).

### 3.2 Descriptive statistics and correlation analysis

Descriptive statistics and correlation coefficients for the variables are presented in **Table 1**. At all time pints, harsh parenting was significantly and positively correlated with shyness(range of *r* = 0.099 to 0.468, all *p*<0.01), harsh parenting was significantly and positively correlated with cyber victimization(range of *r* = 0.099 to 0.468, all *p*<0.01), shyness was positively correlated with cyber victimization(range of *r* = 0.025 to 0.284). In addition, there were significant correlations among harsh parenting at each time point (range of *r* = 0.300 to 0.468, all *p*<0.001) and among shyness at each time point (range of *r* = 0.185 to 0.382, all *p*<0.001) as well as among cyber victimization at each time point(range of *r* = 0.072 to 0.342, all *p*<0.05). Gender was significantly and negatively correlated with T2 harsh parenting(*r* = -0.087, *p* < 0.05) and T2 shyness (*r* = -0.190, *p* < 0.05). Therefore, in subsequent analyses of CLPM and RI-CLPM conducted using Mplus 8.3, we incorporated gender as a control variable in the models.

**Table 1:** Descriptive statistics and correlation analysis (N = 828).

	1	2	3	4	5	6	7	8	9	10
1 T1 Gender	1									
2 T1HP	-0.052	1								
3 T2HP	-0.087^*^	0.426^***^	1							
4 T3HP	0.008	0.300^***^	0.468^***^	1						
5 T1S	-0.026	0.268^***^	0.123^***^	0.099^**^	1					
6 T2S	-0.190^*^	0.268^***^	0.359^***^	0.185^***^	0.382^***^	1				
7 T3S	0.005	0.118^***^	0.106^**^	0.200^***^	0.206^***^	0.185^***^	1			
8 T1CV	-0.007	0.310^***^	0.232^***^	0.167^***^	0.124^***^	0.172^***^	0.042^*^	1		
9 T2CV	-0.047	0.324^***^	0.486^***^	0.180^***^	0.087^*^	0.284^***^	0.103^**^	0.342^***^	1	
10 T3CV	-0.030	0.099^**^	0.187^***^	0.097^**^	0.025	0.202^***^	0.185^***^	0.072^*^	0.223^***^	1
M	1.51	3.740	3.708	3.540	3.553	3.438	3.140	3.267	3.181	3.188
SD	0.5	0.757	0.784	0.766	0.748	0.766	0.702	0.652	0.700	0.860

HP, Harsh Parenting; S, Shyness; CV, Cyber Victimization; T1, Time 1; T2, Time 2; T3, Time 3. Gender is a dummy variable (male = 0, female = 1). ^***^ *p*<0.001;^**^ *p*<0.01;^*^ *p*<0.05.

### 3.3 Longitudinal measurement invariance test

Following established recommendations, the criteria for model fit evaluation include: Tucker-Lewis Index(TLI) > 0.95, Comparative Fit Index(CFI) > 0.9, Standardized Root Mean Square Residual (SRMR) < 0.08, and Root Mean Square Error of Approximation(RMSEA) < 0.06 (57).

Given the longitudinal nature of the data, analyses of longitudinal measurement invariance were conducted. In this study, three levels of measurement invariance were tested across time points: configural invariance, metric invariance (factor loading invariance) and scalar invariance (item intercept invariance). A particular test of equivalence is considered to have been passed if the differences between the individual model fit indicators are less than a set threshold(ΔCFI < 0.01 and ΔRMSEA < 0.015) (58). As presented in **Table 2**, all variables met the criteria for scalar invariance, confirming the robustness of the measurement model across time points.

**Table 2:** Model fit indices for longitudinal measurement invariance analysis.

Model	*χ²*	*df*	CFI	RMSEA (90% CI)	SRMR	Model Comparison	ΔCFI	ΔRMSEA
Harsh Parenting								
M1:Configural Invariance	7.245	6	1	0.016(0.000,0.050)	0.004			
M2:Metric Invariance	17.908	12	0.999	0.024(0.000,0.046)	0.019	M2-M1	0.001	0.008
M3:Scalar Invariance	29.916	18	0.998	0.028(0.007,0.046)	0.021	M3-M2	0.001	0.004
Shyness								
M1:Configural Invariance	174.053	195	1	0.000(0.000,0.009)	0.007			
M2:Metric Invariance	196.769	219	1	0.000(0.000,0.008)	0.016	M2-M1	0	0
M3:Scalar Invariance	234.112	243	1	0.000(0.000,0.012)	0.017	M3-M2	0	0
Cyber Victimization								
M1:Configural Invariance	279.715	231	0.999	0.011(0.006,0.015)	0.008			
M2:Metric Invariance	316.058	257	0.998	0.012(0.007,0.015)	0.019	M2-M1	0	0.001
M3:Scalar Invariance	346.590	283	0.998	0.011(0.006,0.015)	0.020	M3-M2	0	0.001

### 3.4 Cross-lagged analysis of harsh parenting, shyness, and cyber victimization

**Figure 1:**
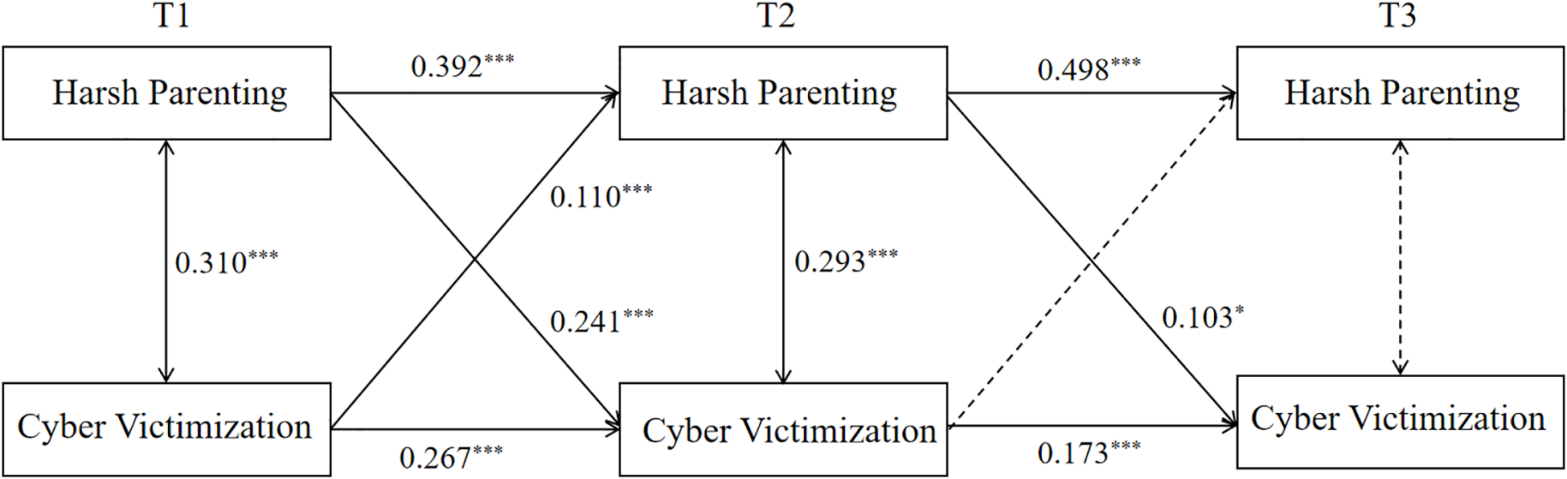
Cross-lagged model of harsh parenting and cyber victimization. Solid lines represent standardized path coefficients that are statistically significant, while dashed lines indicate non-significant coefficients; non-significant paths are omitted from the figure. ***p<0.001; *p<0.05. The same notation convention applies the subsequent figures.

Structural Equation Modeling (SEM) was employed to construct CLPM examining the relationship between harsh parenting and cyber victimization. The model fit indices were as follows: χ²/*df* = 4.373, RMSEA = 0.064, SRMR = 0.025, CFI = 0.974, TLI = 0.907, indicating an acceptable fit to the data. As can be seen in **Figure 1**, T1 harsh parenting positively predicted T2 harsh parenting (*β* = 0.392, *p* < 0.001), and T2 harsh parenting positively predicted T3 harsh parenting (*β* = 0.498, *p* < 0.001). T1 cyber victimization positively predicted T2 cyber victimization (*β* = 0.267, *p* < 0.001), and T2 cyber victimization positively predict T3 cyber victimization(*β* = 0.173, *p* < 0.001). T1 harsh parenting positively predicted T2 cyber victimization (*β* = 0.241, *p* < 0.001), T2 harsh parenting positively predicted T3 cyber victimization (*β* = 0.103, *p* < 0.05), and T1 cyber victimization positively predicted T2 harsh parenting (*β* = 0.110, *p* < 0.001).

**Figure 2:**
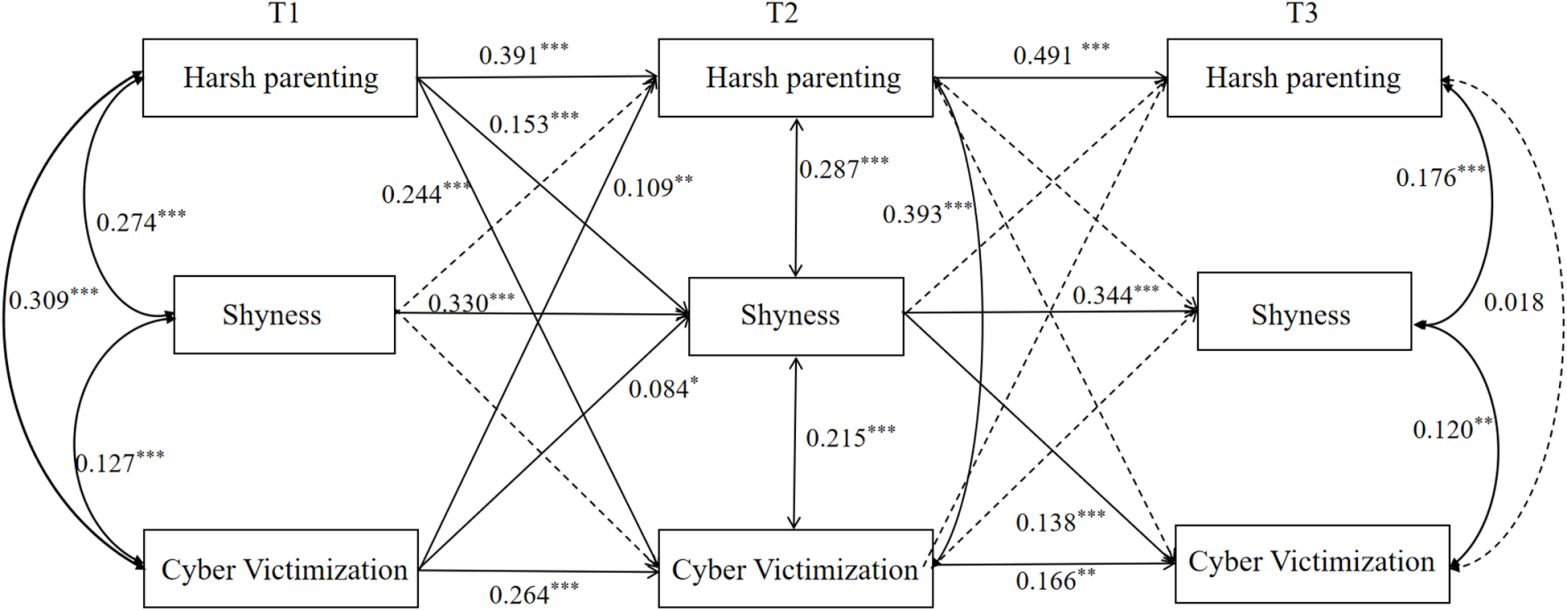
Cross-lagged model of harsh parenting, shyness, and cyber victimization. ***p<0.001;**p<0.01;*p<0.05.

Second, CLPM was employed to examine the relationships among harsh parenting, shyness and cyber victimization. **Figure 2** presents the structural model with standardized path coefficients. The model demonstrated an acceptable fit to the data, as indicated by the following fit indices: χ²/*df* = 3.146, RMSEA = 0.051, SRMR = 0.018, CFI = 0.985, TLI = 0.905. After controlling for gender, T1 harsh parenting positively predicted T2 shyness (*β* = 0.153, *p* < 0.001) and T2 cyber victimization (*β* = 0.244, *p* < 0.01). Additionally, T2 shyness positively predicted T3 cyber victimization (*β* = 0.138, *p* < 0.001). Furthermore, T1 cyber victimization positively predicted T2 harsh parenting (*β* = 0.109, *p* < 0.01) and T2 shyness (*β* = 0.084, *p* < 0.05). Mediation analyses using deviation correction percentile bootstrap method revealed that T2 shyness mediated the relationship between T1 harsh parenting and T3 cyber victimization, with a mediation effect value of 0.021, 95% *CI* [0.011, 0.038].

### 3.5 Random intercept cross-lagged analysis of harsh parenting, shyness, and cyber victimization

**Figure 3:**
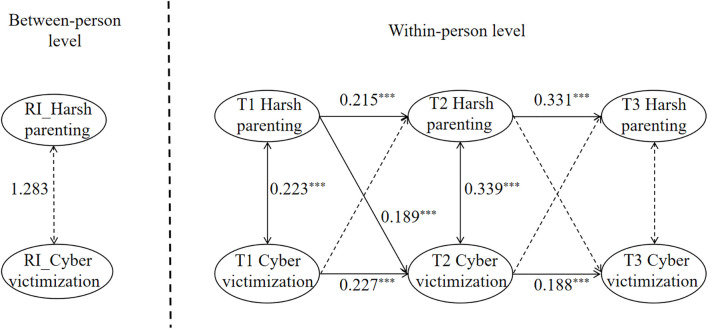
Random intercept cross-lagged panel model of harsh parenting and cyber victimization. RI denotes random intercept. The same notation convention applies to subsequent figures. ***p<0.001.

The relationship between harsh parenting and cyber victimization was analyzed using the random intercept cross-lagged analysis. The model fit indices were as follows: χ²/*df* = 2.739, RMSEA = 0.046, SRMR = 0.014, CFI = 0.998, TLI = 0.930, indicating an acceptable fit to the data. As illustrated in **Figure 3**, the analysis revealed distinct patterns at different levels. At the between-person level, harsh parenting showed no significant association with the random intercept of cyber victimization(*β* = 1.283, *p* > 0.05). At the within-individual level, T1 harsh parenting positively predicted T2 harsh parenting (*β* = 0.215, *p* < 0.001), T2 harsh parenting positively predicted T3 harsh parenting (*β* = 0.331, *p* < 0.001), T1 cyber victimization positively predicted T2 cyber victimization (*β* = 0.227, *p* < 0.001), T2 cyber victimization positively predicted T3 cyber victimization (*β* = 0.188, *p* < 0.001), and T1 harsh parenting positively predicted T2 cyber victimization (*β* = 0.189, *p* < 0.001).

**Figure 4:**
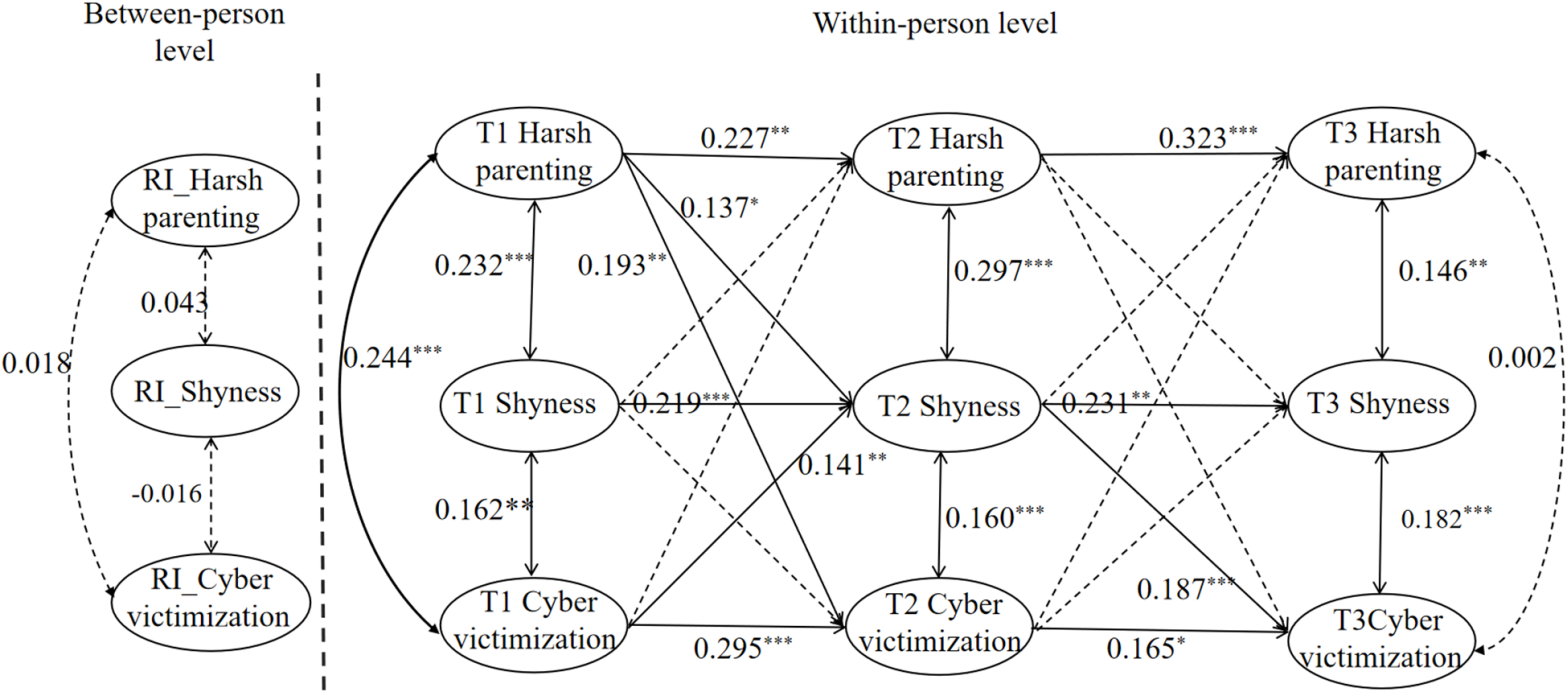
Random intercept cross-lagged panel model of harsh parenting, shyness, and cyber victimization. ***p<0.001;**p<0.01;*p<0.05.

**Figure 4** presents the random intercept cross-lagged analysis examining the relationships among harsh parenting, shyness, and cyber victimization. The model fit indices were as follows: χ²/*df* =1.510, RMSEA = 0.025, CFI = 0.999, TLI = 0.976, SRMR = 0.009, indicating an acceptable fit to the data. At the between-individual level, the random intercepts of harsh parenting, shyness, and cyber victimization were not significantly related. At the within-individual level, T1 harsh parenting positively predicted T2 harsh parenting (*β* = 0.227, *p* < 0.01), T2 harsh parenting positively predicted T3 harsh parenting (*β* = 0.323, *p* < 0.001). T1 shyness positively predicted T2 shyness (*β* = 0.219, *p* < 0.001), T2 shyness positively predicted T3 shyness (*β* = 0.231, *p* < 0.01). T1 cyber victimization positively predicted T2 cyber victimization (*β* = 0.295, *p* < 0.001) and T2 cyber victimization positively predicted T3 cyber victimization (*β* = 0.165, *p* < 0.05). T1 harsh parenting positively predicted T2 shyness (*β* = 0.137, *p* < 0.05) and T2 cyber victimization (*β* = 0.193, *p* < 0.01). T2 shyness positively predicted T3 cyber victimization (*β* = 0.187, *p* < 0.001), and T1 cyber victimization positively predicted T2 shyness (*β* = 0.141, *p* < 0.01). Furthermore, mediation analysis revealed that T2 shyness mediated the relationship between T1 harsh parenting and T3 cyber victimization. The standardized indirect effect from T1 harsh parenting to T3 cyber victimization was significant, with an estimate of 0.026, 95% *CI* [0.007, 0.058]. This suggests that harsh parenting has a positive and statistically significant indirect effect on cyber victimization.

## 4 Discussion

The current study employed both CLPM and RI-CLPM to examine the underlying mechanisms between harsh parenting and cyber victimization, while investigating the mediating role of shyness in this relationship. The results revealed notable differences in the relationships between variables as demonstrated by the two models. The primary reason for the discrepancy in results between these two statistical models lies in their differing interpretations of variable relationships. The CLPM captures an overall association between variables, integrating within-individual and between-individual effects. However, its inability to disentangle these effects can lead to confounding by individual characteristics and measurement errors, thereby obscuring precise insights into variable relationships. In contrast, RI-CLPM explicitly differentiates between within-individual and between-individual effects.

Given the limitations of the CLPM, this study prioritizes the RI-CLPM as the primary analytical framework to separately examine between-individual and within-individual effects. By employing this differentiated approach, we can more precisely analyze the complex relationships between variables. Furthermore, the discrepancy between the results of the CLPM and RI-CLPM offers valuable insights for researchers. This phenomenon underscores the importance of fully considering the inherent limitations of the CLPM when interpreting its findings. Such caution is essential to ensure the accuracy and reliability of research conclusions and to avoid misinterpreting phenomena due to the model's intrinsic flaws.

### 4.1 Inconsistency between CLPM and RI-CLPM results

In this study, we employed the CLPM and RI-CLPM to examine the bidirectional associations between harsh parenting and cyber victimization, as well as the mediating role of shyness. The inconsistency of the results presented by these two statistical analysis models is primarily attributed to the distinct meanings and assumptions inherent to each model.

First, this study examined the bivariate relationship between harsh parenting and cyber victimization. In CLPM, harsh parenting at the previous time point significantly and positively predicted cyber victimization at the later time point. In contrast, in RI-CLPM, harsh parenting was not significantly related to cyber victimization in terms of between-individual effects. Regarding within-individual effects, harsh parenting was only able to significantly and positively predict cyber victimization in the earlier part of the study, and this relationship did not hold in the later part of the study. Second, this study examined the bidirectional mediating role of shyness between harsh parenting and cyber victimization. In CLPM, shyness mediated longitudinally between T1 harsh parenting and T3 cyber victimization. However, in the RI-CLPM, there was no significant correlation among the three at the between-individual effect. At the within-individual level, shyness mediated longitudinally between T1 harsh parenting and T3 cyber victimization. The inconsistency between the CLPM and RI-CLPM results suggests a within-individual effect as the critical pathway for harsh parenting to influence cyber victimization, a result that reflects developmental theories in which the relationship between the stateful components of variables at the within-individual level is at the heart of the research (45).

As the CLPM is recognized as a powerful method for exploring dynamic relationships between variables, the RI-CLPM is generated by adding one or more random intercepts to the CLPM (59). Utilizing these two models can provide a more comprehensive analytical perspective, and thus reveal the interactions between variables more fully (47, 48). Some researchers have found that for the same tracking data, different models will provide different types of parameter information, CLPM and RI-CLPM may differ in their approach to exploring the role between variables (59). Therefore, despite the limitations of the CLPM, we likewise constructed the CLPM model in our study. This approach allows us to more thoroughly explore the research results and better grasp the essence of the relationships between variables. Moreover, by comparing the results of the study, it can provide some insights into how researchers understand and view the two methods.

### 4.2 Harsh parenting and cyber victimization

The relationship between harsh parenting and cyber victimization was differentially reflected at the between-individual level and the within-individual level.

According to the CLPM results, harsh parenting and cyber victimization were mutually predictive in the pre-study period (T1-T2). This result is consistent with the parental acceptance and rejection theory that adolescents who experience harsh parenting will lack protection and support from their parents and are more likely to experience cyber victimization (18, 60). After adolescents experience cyber victimization, there may be negative consequences that force parents to perpetuate harsh parenting (61). In the later part of the study (T2-T3), only harsh parenting significantly and positively predicted cyber victimization, which may be due to the fact that in the later part of the study, when adolescents spend more time in school and less time with their parents, the parents may have lacked awareness of their adolescents' cyber victimization behaviors, which led to a weakening of the effect of cyber victimization on harsh parenting.

Based on RI-CLPM results. First, at the within-individual level, harsh parenting significantly predicted cyber victimization at the T1-T2 stage, but not at the T2-T3 stage. These findings align with social learning theory, demonstrating that adolescents who are victims of harsh parenting acquire this pattern of “victimization” and are able to accept and internalize the violence, resulting in greater acceptance of cyber victimization (21). This result can be explained by the life history theory, in which individuals develop certain adaptive strategies in the face of adversity (e.g., harsh parenting), and these adaptive strategies promote the development of the individual in unfavorable conditions (62). From this perspective, adolescents who are exposed to harsh parenting early in life are susceptible to its effects due to a lack of coping styles. Over time, some adolescents may acquire coping strategies, and in the later stages of the study, when adolescents are entering the stage of school entrance exams, schools tighten their control over adolescents' online behaviors, which reduces the incidence of cyber victimization to a certain extent. Adolescents who suffer from harsh parenting in the early stages are vulnerable to its effects due to a lack of coping styles. Over time, some adolescents may acquire coping strategies, and in the later stages of the study, when adolescents enter the stage of college entrance exams, schools will strengthen the control of adolescents' online behaviors, which will reduce the occurrence of cyber victimization to a certain extent. It is worth noting that the role of cyber victimization on harsh parenting was not significant. This may be related to the hidden nature of cyber victimization behavior (63). And compared to the visible physical scars or emotional damage in traditional victimization, cyber victimization may be difficult for parents to perceive (9).

Finally, the study found that at the between-individual level, harsh parenting was not significantly associated with cyber victimization. Since the between-individual level responds to an individual's trait component that is stable on the variable (59), this finding suggests that harsh parenting is not associated with the stable trait of cyber victimization. That is, the group of adolescents with high levels of harsh parenting also did not necessarily have higher levels of cyber victimization throughout the study period. According to ecosystem theory, individuals' development is influenced by multiple levels of environmental systems, and harsh parenting belongs to the family factors in the microsystem (64). During the study period, adolescents were in the school environment, and their cyber victimization was also influenced by multiple factors such as school and peer relationships in the microsystem (64). These factors interacted with each other and may have led to the inability to show a significant correlation between harsh parenting and cyber victimization at the between-individual level.

### 4.3 Longitudinal mediation of shyness

The study revealed relative stability in adolescents' experiences of harsh parenting, shyness, and cyber victimization across the three time points at the within-individual level. Harsh parenting, shyness, and cyber victimization in the previous period all significantly predicted the corresponding variables in the later period.

Furthermore, both the CLPM and RI-CLPM results found that T2 shyness mediated the relationship between T1 harsh parenting and T3 cyber victimization. Parental acceptance-rejection theory can effectively explain the relationship between harsh parenting and shyness. When individuals experience lower levels of parental support and parental warmth, it makes adolescents feel that their emotions and needs are not sufficiently valued and satisfied, which makes them prone to feelings of shame and inferiority, and increases the probability of their shyness behaviors (33, 60). Such behavioral patterns manifest not only in face-to-face interactions but also extend to online environments, negatively impacting adolescents' digital social experiences. On the one hand, in online social contexts, shy individuals typically exhibit reduced self-disclosure tendencies and greater reluctance to share personal information, emotions, and thoughts. This behavioral pattern may result in social misinterpretation or neglect from peers, thereby elevating their vulnerability to cyber victimization (65). This shy behavior not only affects real-world socialization, but also extends to the online environment, negatively impacting adolescents' online socialization. On the other hand, shy individuals' lack of social support makes it difficult for them to get timely help when experiencing cyber victimization, thus increasing their vulnerability (37). Moreover, the anonymity and wide reach of online platforms lower the threshold for bullying behaviors, increasing shy adolescents' risk of victimization (2). When facing negative online interactions, these individuals often demonstrate limited coping efficacy due to insufficient social support. This phenomenon not only reveals the special dilemma of shy individuals in online environments, but also illustrates the mechanism of interaction between online and real socialization in bullying behavior.

### 4.4 Research significance

In terms of theoretical implications, this study systematically examined the relationship between harsh parenting, shyness, and cyber victimization by constructing the CLPM and RI-CLPM. From the perspective of developmental contextualism, the study suggests that cyber victimization of adolescents does not result from steady inter-individual trait differences, but is closely related to real-time fluctuations. Short-term changes in harsh parenting (e.g., a spike in parent-child conflict within a given period) may trigger immediate shyness emotional responses (e.g., heightened tendency to socially withdraw) in adolescents, which in turn increases their risk of experiencing cyber victimization within the same period. The findings provide a new theoretical perspective for understanding the dynamic mechanisms of adolescent psychological adjustment, which challenges the static assumptions of traditional cross-sectional studies that attribute variable associations to group stability traits (44). In addition, the findings highlight the importance of distinguishing between within-and between-individual effects in theory construction, whereas traditional CLPM analyses may overestimate the long-term predictive role of variables (e.g., harsh parenting) by confounding different levels of effects, the RI-CLPM analyses suggest that the effects of harsh parenting on cyber victimization are more reflective of short-term paths of action. The results from this study using the RI-CLPM emphasize the importance of considering both within-and between-individual effects when addressing the direct or indirect bi-directional associations between harsh parenting and cyber victimization among adolescents in the future.

The findings provide important implications for addressing adolescent cyber victimization. At the family level, parents should adopt constructive parenting practices that emphasize open communication and promote positive self-evaluation in adolescents. Schools can support this by offering parent education programs to reduce harsh parenting and encourage positive alternatives. Additionally, schools and communities should collaborate to develop digital literacy initiatives, incorporating diverse activities to enhance adolescents' awareness of online risks. Ultimately, a coordinated approach involving families, schools, and communities, supported by early intervention and long-term monitoring, is essential for effective prevention and intervention.

### 4.5 Limitations

This study has several limitations that warrant consideration. First, the reliance on self-reported data may introduce recall bias and social desirability effects, potentially leading to discrepancies between adolescents' perceptions of harsh parenting and actual parenting behaviors. Future research could enhance data reliability by incorporating multiple methodologies, such as experimental designs and in-depth interviews. Second, the sample was limited to students from four middle schools in Anhui Province, and the 16-month longitudinal design may not fully capture long-term dynamics between variables. Expanding the sample across regions and school types, along with a longer study duration, would strengthen the findings' applicability and provide deeper insights into longitudinal relationships. Finally, there may be other mediating or moderating mechanisms in the relationship between harsh parenting and cyber victimization, and this study only explored the mediating role of shyness between the two. Therefore, other possible variables can be explored in future studies to enrich the research between the two.

## 5 Conclusions

The findings of this study are as follows: (a) In CLPM, T1 and T2 harsh parenting significantly and positively predicted cyber victimization at the latter time point, harsh parenting and cyber victimization at T1 and T2 had reciprocal predictive effects. (b) In RI-CLPM, at the between-individual level, harsh parenting was not significantly positively correlated with cyber victimization; at the within-individual level, T1 harsh parenting significantly positively predicted T2 cyber victimization. (c) In CLPM and RI-CLPM, T2 shyness mediated longitudinally between T1 harsh parenting and T3 cyber victimization. The stability of harsh parenting, shyness and cyber victimization was high during the 16 months of the study.

The present study uses both CLPM and RI-CLPM models to explore the relationship among harsh parenting, shyness and cyber victimization, which is beneficial to enriching the relevant theories and deepening the understanding of the dynamic relationship among the variables. Also, it is conducive to providing important practical guidance for the intervention of cyber victimization among adolescents and drawing the attention of families and schools to related issues.

## Data Availability

The raw data supporting the conclusions of this article will be made available by the authors, without undue reservation.
